# Personalized Porous Gelatin Methacryloyl Sustained‐Release Nicotinamide Protects Against Noise‐Induced Hearing Loss

**DOI:** 10.1002/advs.202305682

**Published:** 2024-01-15

**Authors:** Baoyi Feng, Tingting Dong, Xinyu Song, Xiaofei Zheng, Chenxi Jin, Zhenzhe Cheng, Yiqing Liu, Wenjie Zhang, Xueling Wang, Yong Tao, Hao Wu

**Affiliations:** ^1^ Department of Otolaryngology‐Head and Neck Surgery Shanghai Ninth People's Hospital Shanghai Jiao Tong University School of Medicine No.639, Zhizaoju Road Shanghai 200011 P. R. China; ^2^ Ear Institute Shanghai Jiao Tong University School of Medicine No.115, Jinzun Road Shanghai 200125 P. R. China; ^3^ Shanghai Key Laboratory of Translation Medicine on Ear and Nose Disease No.115, Jinzun Road Shanghai 200125 P. R. China; ^4^ Biobank of Ninth People's Hospital Shanghai Jiao Tong University School of Medicine No.115, Jinzun Road Shanghai 200125 P. R. China; ^5^ Department of Prosthodontics Shanghai Ninth People's Hospital Shanghai Jiao Tong University School of Medicine Shanghai 200011 P. R. China

**Keywords:** hearing loss, NAD, noise, personalized delivery, porous gelatin methacryloyl

## Abstract

There are no Food and Drug Administration‐approved drugs for treating noise‐induced hearing loss (NIHL), reflecting the absence of clear specific therapeutic targets and effective delivery strategies. Noise trauma is demonstrated results in nicotinamide adenine dinucleotide (NAD+) downregulation and mitochondrial dysfunction in cochlear hair cells (HCs) and spiral ganglion neurons (SGNs) in mice, and NAD+ boosted by nicotinamide (NAM) supplementation maintains cochlear mitochondrial homeostasis and prevents neuroexcitatory toxic injury in vitro and ex vivo, also significantly ameliorated NIHL in vivo. To tackle the limited drug delivery efficiency due to sophisticated anatomical barriers and unique clearance pathway in ear, personalized NAM‐encapsulated porous gelatin methacryloyl (PGMA@NAM) are developed based on anatomy topography of murine temporal bone by micro‐computed tomography and reconstruction of round window (RW) niche, realizing hydrogel in situ implantation completely, NAM sustained‐release and long‐term auditory preservation in mice. This study strongly supports personalized PGMA@NAM as NIHL protection drug with effective inner ear delivery, providing new inspiration for drug‐based treatment of NIHL.

## Graphical abstract

NAD+ decreases in cochlear after noise trauma, and nicotinamide supplementation boosts NAD+ and prevents neuroexcitatory in cochlear by round window administration. Personalized porous gelatin methacryloyl (PGMA) achieves long term drug release in cochlear, and nicotinamide encapsulated PGMA enhances mitochondria and synapse function, and protects noise induced hearing loss in mice.

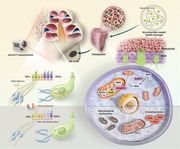

## 1 Introduction

It has been estimated that 430 million people suffer from moderate or higher levels of hearing loss,^[^ ^1^ ^]^ establishing hearing loss as a worldwide public health challenge. Noise is the main cause of deafness, and NIHL is the second‐most prevalent type of sensorineural hearing loss.^[^ ^2^ ^]^ Increasing numbers of adolescents are being diagnosed with extended high‐frequency hearing loss owing to prolonged, daily acoustic exposure.^[^ ^3^ ^]^ Notably, this population, has now overtaken those diagnosed with occupational NIHL (≈16%).^[^ ^4^ ^]^ Transient blasting and continuous loud sounds can damage the vulnerable structures of the inner ear, impairing auditory function and increasing the risk of speech‐recognition disorders.^[^ ^5^ ^]^ Noise trauma can cause transient hearing loss or a permanent threshold shift (PTS) and thus represents a serious health hazard.

Extensive studies have investigated the contributions of a number of molecular mechanisms to NIHL, including oxidants and free radical scavengers,^[^ ^6^ ^]^ reactive oxygen species (ROS)‐sensitive pathways,^[^ ^7^ ^]^ and inflammatory,^[^ ^8^ ^]^ autophagy^[^ ^9^ ^]^ and cell‐death pathways.^[^ ^10^ ^]^ Noise‐induced synapses degeneration has been studied, and synaptopathy is a major reason for NIHL.^[^ ^11^ ^]^ However, the mechanisms crucial to the development of NIHL remain uncertain, posing a major barrier to NIHL treatment.

Mitochondria dominate cellular energy metabolism and ATP production, and are responsible for regulating the dynamic equilibrium between calcium and apoptosis.^[^ ^12^ ^]^ Mitochondrial dysfunction and mitochondrial DNA (mtDNA) mutations are known to be associated with sensorineural hearing loss,^[^ ^13^ ^]^ and their roles in NIHL have recently been illustrated. Upon exposure to high‐intensity noise, injured dysfunctional mitochondria release massive amounts of ROS into the endoplasm of cochlear cells, including HCs and cells of the stria vascularis.^[^ ^2^, ^14^ ^]^ Moreover, ATP depletion in the inner ear is observed in the hours following noise exposure.^[^ ^15^ ^]^ ROS release and ATP consumption further activate an AMPKα‐mediated pathway that leads to outer hair cell (OHC) death and auditory neuropathy.^[^ ^16^ ^]^ Given that mitochondrial function is thought to play an essential role in NIHL pathology, an array of molecules known to enhance mitochondrial function^[^ ^17^ ^]^ have been studied for their potential to alleviate NIHL, including sirtuin mediators,^[^ ^18^ ^]^ coenzyme Q10 and its analog^[^ ^19^ ^]^ and CDK2 inhibitors.^[^ ^20^ ^]^ Although none of the numerous prospective therapeutic molecules investigated to date has been approved for clinical use in patients with NIHL,^[^ ^17^ ^]^ enhancing mitochondrial function remains a promising potential treatment for NIHL.

RNAD^+^, a redox reaction coenzyme and cofactor of NAD^+^‐dependent enzymes, including PRAP1 and sirtuins, plays essential roles in cellular metabolism and also participates in mitochondrial functions, DNA repair, inflammation, and immune responses.^[^ ^21^ ^]^ NAD^+^ depletion has been implicated in multisystem age‐related diseases, such as neurodegeneration, diabetes, cancer, and cardiovascular disease.^[^ ^22^ ^]^ Because mammalian cells are unable to directly take up NAD^+^, mouse models of human diseases have widely employed NAD^+^‐boosting strategies, including inhibition of NAD^+^ degradation and activation of NAD^+^ biosynthetic enzymes.^[^ ^23^ ^]^ In this latter context it has been reported that augmentation of NAD^+^ through the enzymatic action of NAD(P)H quinone oxidoreductase‐1 (NQO1) ameliorates age‐related hearing loss (ARHL) and cisplatin‐induced hearing impairment in mice.^[^ ^24^ ^]^ Although NAD^+^‐mediated sirtuin 3 (Sirt3) activation has been shown to protect against NIHL,^[^ ^18^ ^]^ NAD^+^ function in NIHL has not been studied.

An important NAD^+^‐boosting strategy is supplementation of NAD^+^ precursors, which include NAM, nicotinamide mononucleotide (NMN), nicotinamide riboside (NR), and niacin (NA). NMN supplementation has been shown to improve glucose intolerance,^[^ ^25^ ^]^ preserve mitochondrial homeostasis in aging mice,^[^ ^26^ ^]^ and protect against cisplatin‐induced hearing loss.^[^ ^27^ ^]^ Increasing NAD^+^ by administering NR was also shown to prevent NIHL and ARHL in rodents,^[^ ^18^, ^28^ ^]^ but the instability of NR in the circulation complicates its delivery and limits its treatment efficacy, which might be the reason for suboptimal clinical efficacy in human with oral NR supplementation.^[^ ^29^ ^]^ Another potential NAD^+^ booster with potential efficacy in treating NIHL is NAM, which is both an NAD^+^ precursor and a byproduct of NAD^+^ consumption. Chronic dose‐appropriate supplementation of NAM, which can be converted to NAD^+^ through the salvage pathway, has been shown to improve healthspan^[^ ^30^ ^]^ and ameliorate neurodegeneration in the central and peripheral nervous systems.^[^ ^31^ ^]^ However, to date, the efficacy of NAM in the treatment of deafness has not been studied.

The cochlea, an isolated hearing organ within the inner ear, is surrounded by the blood‐labyrinth barrier (BLB), which forms a physiological barrier that prevents drug dispersal to the cochlea.^[^ ^32^ ^]^ In one study, the concentration of drug in the blood was reported to be 92000‐times higher than that in the cochlea in rodents following systemic administration,^[^ ^33^ ^]^ indicating that systemic administration route is not a viable option for delivering drugs to the inner ear. The most effective reported method for treating deafness is local delivery of drugs into the inner ear through the round window membrane (RWM),^[^ ^34^ ^]^ located at the bottom of the RW niche. The RW niche, which consists of the bony pouch of the tympanic cavity, is a natural space for a drug‐delivery system.

The efficacy of local administration into the inner ear depends on the size of the contact “footprint” between the delivery system and the RWM. The size of this footprint is, in turn, determined by topographical constraints and the effectiveness of RWM contact. Injectable hydrogels have been shown to support RWM adhesion,^[^ ^35^ ^]^ but the volume of material that can be applied is limited and the approach is operationally inconvenient. Exploring the topography of the RW niche and constructing a framework that perfectly fits it could improve drug‐delivery efficiency, but data from murine models are ambiguous. Therefore, we reconstructed the RW niche cavity based on micro‐CT‐assisted topography and printed a custom‐fit delivery system via 3D‐printing technology. Based on this system, personalized drug delivery vehicles can be generated for inner ear treatment. Gelatin methacryloyl (GelMA), a biocompatible hydrogel that can be easily shaped and rapidly polymerizes under ultraviolet (UV) irradiation, has been applied in acquired deafness. Serving as a drug carrier, GelMA not only displays biocompatibility and tunable mechanical features, but also can adhere to the RWM for a long period of time to achieve higher concentration of drugs in the perilymph.^[^ ^36^ ^]^ Nonetheless, when the gel is directly applied to the middle ear cavity, it appears in bulk form without pores, which tends to interfere with sound transmission. To overcome this problem, GelMA microgel particles generated by microfluidic technology are developed, which allow sound conduction through the interspace between these microshperes.^[^ ^37^ ^]^ However, these microspheres can only adhere to the surface of RWM in a point‐contact manner, so the limited contact area will reduce the release rate of drugs and their therapeutic effects. Due to the unique physiological function of the inner ear, the biomaterial administrated locally needs to be fully contacted with the RWM and do not interfere the sound transmission. GelMA with porous structures generated from personalized delivery system might meet these needs because of enough contact areas and pores allowing sound conduction. However, the safety and efficacy of personalized porous GelMA have not yet been studied.

In this study, we sought to characterize the effects of NAM supplementation and optimize a sustained‐release system to protect against NIHL in mice. Starting by investigating the effects of noise exposure on mitochondrial function and NAD^+^ levels, we found that noise trauma impacted mitochondria of HCs and SGNs. We subsequently found that NAM supplementation was capable of maintaining mitochondrial homeostasis and protecting cochleae from neuroexcitatory toxic injury in vitro and in vivo. To generate a porous structure that allowed air conduction of sound, we optimized NAM‐encapsulated GelMA through lyophilization. Importantly, we constructed and printed a drug‐delivery system tailored to the specific RW niche structure of the mouse that released drugs efficiently. The resulting personalized porous GelMA (PGMA) exhibited a large area of contact with the irregularly shaped RWM, allowing sustained drug release without interfering with sound transmission. NAM delivered via PGMA (NAM@PGMA) demonstrated the capacity for long‐term release through the RWM and prevented noise‐induced mitochondrial and synapse degeneration. Moreover, NAM@PGMA persistently and effectively protected against NIHL by enhancing mitochondrial homeostasis and synapse regeneration, suggesting a potential treatment for individuals who are susceptible to or suffer from the impacts of noise.

## 2 Results

### 2.1 Mitochondrial Dysfunction and NAD^+^ Depletion in Murine Cochleae After Noise Trauma

**Figure 1:**
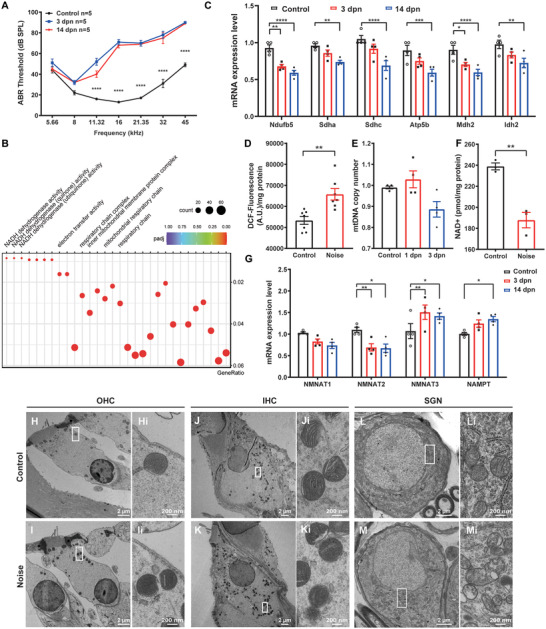
Mitochondrial dysfunction and NAD^+^ depletion during NIHL. A) ABR threshold of all frequencies of the mice without noise exposure or at 3, 14 dpn (*n* = 5 mice). B) GO enrichment analysis of downregulated DEGs in the cochleae before noise and 1 dpn. The top 20 GO pathways enriched at each group were shown. Size of the black circles indicates the gene numbers, while the color of each dot represents the corrected *P*‐adjust for the corresponding terms (*n* = 4 from 4 cochleae). C) mRNA levels of the genes relative to OXPHOS including *Ndufb5*, *Sdha*, *Sdhc*, *Atp5b*, and TCA including *Mdh2* and *Idh2* were determined by qRT‐PCR in the cochleae of mice before noise or at 3,14 dpn (*n* = 3 cochleae). D) ROS level measured by microplate reader using DCFH‐A dye and normalized by protein level in the cochleae of mice before or 4 h after noise (*n* = 6 cochleae). E) mtDNA copy number in the cochleae of mice before noise or at 1, 3 dpn (*n* = 4 cochleae). F) NAD^+^ level in cochlea was obtained from mice before noise or at 1 dpn (*n* = 3 cochleae) and normalized by protein level. G) mRNA expression level of *Nmnat1*, *Nmnat2*, *Nmnat3*, *Nampt* in cochleae were determined without noise exposure or at 3, 14 dpn (*n* = 4 mice). H–M) Representative transmission electron microscopic images of mitochondria in OHC, IHC, and SGN in the cochleae of the mice before noise and 1 dpn. Data were analyzed by t test, one‐way or two‐way ANOVA and represent as mean ± SEM. *P < 0.05, **P < 0.005, ***P < 0.0005, ****P < 0.0001.

To determine the primary reason for cochlear damage after noise trauma, we first exposed adult (4‐5 weeks old) C57BL/6J mice to an 8–16 kHz octave band noise at 104 dB SPL (sound pressure level) for 2 h. HCs, SGNs, and their connections are crucial components of the auditory pathway. Here, we found that MYO7A‐labeled HCs and their connections to SGNs (labeled for neurofilament H [NF‐H]) were unchanged in noise‐exposed cochleae (Figure S1A–C, Supporting Information) at 3 or 14 days post‐noise exposure (dpn). However, noise exposure resulted in permanent ABR (auditory brainstem response) threshold shifts of 30 to 60 dB across the range of 11.32 to 45 kHz at 3 and 14 dpn (**Figure** **1**A). To further explore the molecular basis of the effect of noise trauma, we performed an RNA sequencing (RNAseq) study and Gene Ontology (GO) enrichment analysis of differentially expressed genes (DEGs) in the cochleae between mice with and without noise exposure at 1 dpn. The top 20 downregulated gene‐enriched GO terms (Figure 1B) were mainly related to NADH dehydrogenase and the mitochondrial respiratory chain, revealing an association between noise exposure and abnormal NAD^+^ metabolism induced mitochondrial dysfunction. To confirm that mitochondrial function was altered after noise trauma, we assessed the expression levels of the oxidative phosphorylation (OXPHOS) and trichloroacetic acid (TCA) cycle‐related genes, *Ndufb5*, *Sdha*, *Sdhc*, *Atp5b*, *Mdh2*, and *Idh2*, by quantitative reverse transcription‐polymerase chain reaction (qRT‐PCR). This analysis showed that these genes were decreased in mouse cochleae at both 3 and 14 dpn (Figure 1C). Thus, the results of our RNAseq and qRT‐PCR analyses are consistent with the presence of mitochondrial dysfunction in mouse cochleae after noise trauma. To further assess energy metabolism aspects of mitochondrial function, we measured ROS levels in cochleae by monitoring the fluorescence intensity of the redox probe, 2′,7′‐dichlorodihydrofluorescein diacetate (DCFH‐DA). Acoustic stimulation significantly enhanced DCFH‐DA intensity, increasing it by 23.3% ± 6.2% compared with that in non‐noise–exposed cochleae (Figure 1D). mtDNA copy number, measured as a proxy for mitochondrial number, was not changed in cochleae exposed to acoustic trauma, as assessed at 1 and 3 dpn (Figure 1E). NAD^+^ depletion in cochleae was observed at 1 dpn, and expression of *Nmnat1* and *Nmnat2* mRNA, encoding NAD+ biosynthetic enzymes, progressively declined at 3 and 14 dpn, suggesting an NAD^+^ metabolic disorder and mitochondrial dysfunction in noise‐exposed murine cochleae (Figure 1F,G). Transmission electron microscopy (TEM) imaging of mitochondrial morphology revealed the presence of injured mitochondria with vacuolar degeneration and broken cristae in inner hair cells (IHCs), SGNs and myelinated nerves at 1 dpn (Figure 1H–M; Figure S1D,E, Supporting Information), suggesting that mitochondria in the main auditory cell types are vulnerable to noise exposure. Taken together, our results showed that noise‐exposed mice exhibit mitochondrial dysfunction and NAD^+^ depletion in cochleae and mitochondrial pathology in HCs and SGNs.

### 2.2 The NAD^+^ Precursor, NAM, Protects Auditory Neurons from Excitatory Toxicity In Vitro

**Figure 2:**
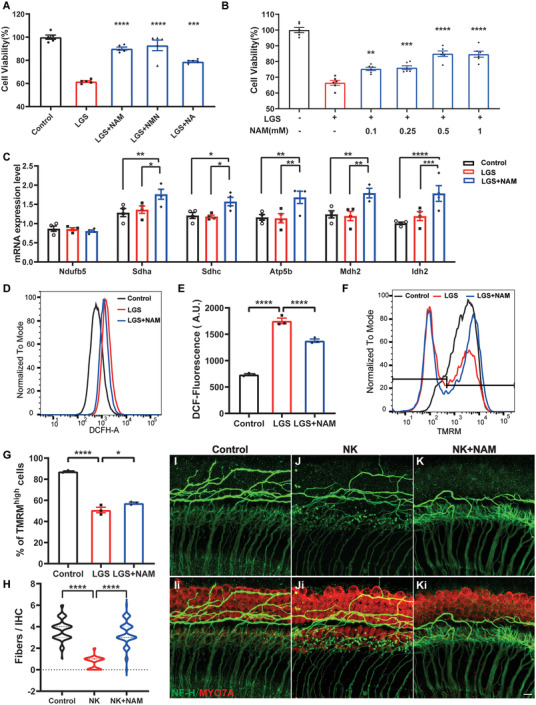
NAM protected Neuro‐2a and cochlear explants from excitotoxicity. A) Cell viability of Neuro‐2a treated in 120 mm LGS with/without 500 um NAM, NMN, NA for 24 h was measured by CCK8 assay (*n* = 5). B) Cell viability of Neuro‐2a treated in 120 mm LGS with various dosage of NAM (*n* = 6). C) mRNA levels of the genes relative to OXPHOS and TCA in Neuro‐2a treated in 120 mm LGS with or without 500uM NAM for 12 h (*n* = 4). D,E) Representative flow cytometric plot and quantification showed the intracellular ROS level of Neuro‐2a treated with LGS and NAM for 12 h. (*n* = 3). F,G) Mitochondrial membrane potential of Neuro‐2a measured by TMRM staining, cells were divided into TMRM^high^ and TMRM^low^ according to TMRM intensity with TMRM^high^ percentage analysis (*n* = 3). H–K) Cochlear explants were stained with Myo7a (Red) to identify hair cells and NF‐H (Green) to label neurofilament of SGNs, the number of the nerve fibers contacting to inner hair cells were calculated (*n* = 60 IHCs from 3 cochlear explants). Scale bars: 10 µm. Data were analyzed by one‐way or two‐way ANOVA and represented as mean ± SEM. *P < 0.05, **P < 0.005, ***P < 0.0005, ****P < 0.0001.

A previous study showed that excitatory receptors activated by released glutamate and glycine are responsible for noise trauma‐induced impairment of cochleae,^[^ ^38^ ^]^ so neural cell line maybe suitable for deafness drug screening. To further study noise‐induced neuropathy, we mimicked noise‐induced hearing loss in vitro by exposing Neuro‐2a cells, a mouse neuroblastoma (neural crest‐derived) cell line, to glutamate excitotoxicity, which causes cell death.^[^ ^39^ ^]^ We then assessed whether NAD^+^ replenishment with NAD^+^ precursors within the NAD^+^ salvage pathway, namely NA, NMN, and NAM, ameliorated this neuroexcitatory toxicity. Treatment with 120 mm L‐glutamate sodium (LGS) reduced the viability of Neuro‐2a cells by 38.3% ± 8.7%, an effect that was markedly mitigated by all three NAD^+^ precursors (applied at 500 µm). Notably, NAM and NMN rescued the viability of Neuro‐2a cells to a greater extent than NA (**Figure** **2**A). NAM and NMN showed similar abilities to protect against neuroexcitatory toxicity, but the protection with NAM (standard error of the mean [SEM] = 1.5%) were less variable than those with NMN (SEM = 4.6%) (Figure 2A). We next evaluated the concentration‐response relationship for NAM‐mediated protection of Neuro‐2a cells against LGS‐induced toxicity, identifying 0.5 mm as the optimal concentration of NAM for use in subsequent studies (Figure 2B). At this concentration, NAM increased the expression of mRNAs for OXPHOS‐ and TCA‐related genes in Neuro‐2a cells 1 day after LGS treatment (Figure 2C). LGS‐induced damage was also associated with a striking increase (138.1% ± 7.0%) in ROS production in Neuro‐2a cells, and the ROS increase that was significantly ameliorated (50.9% ± 7.0% decrease) by NAM treatment (Figure 2D,E). We next assessed the effect of LGS treatment on mitochondrial membrane potential (MMP) by staining cells with tetramethylrhodamine methyl ester (TMRM). LGS treatment decreased the proportion of TMRM^high^ cells (i.e., cells with high MMP) by 36.5% ± 2.3%; this effect was ameliorated by NAM, which reduced this decrease to 6.5% ± 2.3% (Figure 2F,G). In addition to Neuro‐2a cells, we also tested the effects of NAM in the human neuroblastoma cell line, SH‐SY5Y. LGS decreased the viability of SH‐SY5Y cells and significantly increased their ROS levels (by ≈80%). The cell death and mitochondrial dysfunction were reversed by NAM administration (Figure S2A–C, Supporting Information). Mitochondrial morphology was also severely damaged by LGS treatment in both Neuro‐2a and SH‐SY5Y cells, effects that were rescued by NAM (Figure S2D–I, Supporting Information). Collectively, these data indicated that mitochondrial function is enhanced by NAM in vitro. Given that NAM exerted promising effects in controlling mitochondrial function and protecting neural cells against glutamate‐induced cell death, we next evaluated the potential protective role of NAM in cochlear explants. Treatment with 0.5 mm N‐methyl‐D‐aspartate acid (NMDA) and 0.5 mm kainic acid (NK treatment), which triggers severe disconnection of axons from IHCs,^[^ ^40^ ^]^ was used to simulate noise‐induced nerve excitotoxicity ex vivo. As shown in Figure 2H–K, NK treatment resulted in disconnection of axons from IHCs (≈1 fiber/IHC) compared with that observed in untreated cochlea (≈4 fibers/IHC). Notably, NAM restored connections to a level comparable to that in controls (≈4 fibers/IHC), providing the most prominent protective effect among NAD precursors (Figure S3A–E, Supporting Information). These results suggest that NAM provides protection against neural excitotoxicity both in vitro and ex vivo.

### 2.3 NAM, Delivered via a Gelatin Sponge, Prevents Auditory Dysfunction after Noise Exposure in Mice

**Figure 3:**
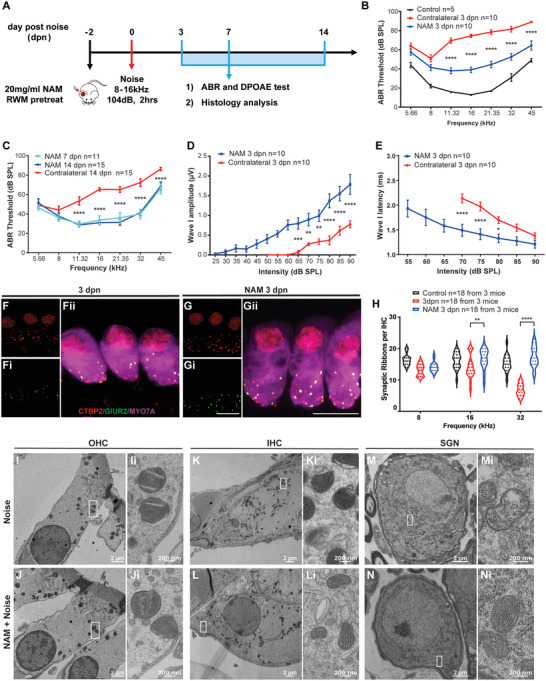
GS delivering NAM protected auditory function from NIHL in vivo. A) Experimental procedure. Adult (P28) C57BL/6J mice were pretreated with 20 mg ml^−1^ NAM on one side of the cochleae through RWM permeation 2 days before noise exposure. ABR and DPOAE test, histology analysis were conducted onto the mice at 3, 7, and 14 dpn. B,C) ABR threshold of all frequencies of the mice with/without NAM delivery at 3, 7, and 14 dpn (*n* = 5–15 mice). D,E) Wave I latency and amplitudes of ABR at 16 kHz of the mice at 3 dpn (*n* = 10 mice). F,G) Representative confocal images stained with Myo7a (Magenta), GLUR2 (Green) and CTBP2 (Red) in the IHCs at 16 kHz of the cochleae at 3 dpn. Scale Bar: 10 µm. H) Bar graphs represented the numbers of synaptic ribbons counting by ctbp2 puncta in each IHC (*n* = 18 IHCs from 3 mice). I–N) Representative TEM images of mitochondria in OHC, IHC and SGN of the mice with/without NAM at 1 dpn. Data were analyzed by two‐way ANOVA and represented as mean ± SEM. *P < 0.05, **P < 0.005, ***P < 0.0005, ****P < 0.0001.

To study the protective efficacy of NAM from NIHL in vivo, we delivered NAM via a NAM‐loaded resorbable gelatin sponge (GS) positioned on the RWM—a traditional method for delivering drugs to the inner ear.^[^ ^41^ ^]^ ABR threshold, distortion product otoacoustic emission (DPOAE), and histologic analyses were conducted at 3, 7, and 14 dpn in mice whose ears were treated with or without NAM 2 days before noise exposure (**Figure** **3**A). At 3 dpn, ABR thresholds were 40–60 dB from 11 to 45 kHz in NAM‐treated cochleae compared with 70–80 dB in untreated contralateral cochleae (Figure 3B; Figure S4A,B, Supporting Information). This change in ABR threshold was maintained up to 14 dpn, whereas the thresholds of GS absorbed with water (sham‐treated) treated cochleae did not differ from those of contralateral ears at 3, 7, or 14 dpn (Figure 3C; Figure S4C, Supporting Information), demonstrating NAM protects against a noise‐induced PTS. We further found that wave I latency was shorter in NAM‐treated ears than contralateral ears at 70, 75, and 80 dB SPL (16 kHz) at 3 dpn, and that wave I amplitude was significantly higher in NAM‐treated ears than contralateral ears at 65–90 dB SPL (Figure 3D,E), indicating that afferent nerve function was protected by NAM treatment. The DPOAE thresholds were 40–60 dB SPL in NAM‐treated ears at 7 and 14 dpn, and 15–23 dB better than those of contralateral ears from 11 to 21 kHz at 14 dpn (Figure S4D–F, Supporting Information), revealing that NAM partially protects OHC motility. We observed a DPOAE threshold increasement after treatment and recovered to normal level 14 dpn, indicating GS treatment insulted outer hair cell function or altered middle ear function temporarily. To examine noise‐induced synaptopathy, we harvested cochleae and stained pre‐synapses for C‐terminal binding protein 2 (CTBP2) and post‐synapses for glutamate receptor 2 (GLUR2). This analysis showed that CTBP2 and GLUR2 were colocalized in NAM‐treated cochleae at both 3 and 14 dpn. A quantification of CTBP2 puncta, taken as representing synapses (Figure 3F,G; S5A‐B, Supporting Information), showed that, at 3 dpn, exposure of contralateral cochleae to noise decreased the number of CTBP2 puncta from 16 to 14 per IHC at 16 kHz, and from 15 to 6 per IHC at 32 kHz; in contrast, the number of puncta at presynapses was preserved in NAM‐treated cochleae (17 and 18 per IHC at 16 and 32 kHz, respectively) (Figure 3H). At 14 dpn, there were 6 additional presynaptic puncta per IHC in treated than contralateral cochleae at 16 kHz and 8 more at 32 kHz (Figure S5C, Supporting Information). After NAM treatment, healthy mitochondria were observed in OHCs, IHCs, SGNs, and myelinated nerves. NAM appeared to be particularly protective against noise‐induced damage of mitochondrial structures in SGNs and myelinated nerves, as evidenced by the relative lack of swollen and/or broken mitochondrial cristae at 1 dpn (Figure 3I–N; Figure S5D,E, Supporting Information). GS‐mediated local administration of NAM partially protected against NIHL, as well as synapse and mitochondria degeneration.

### 2.4 GS‐Delivered NAM Protects Mitochondrial Homeostasis Against Noise Trauma

**Figure 4:**
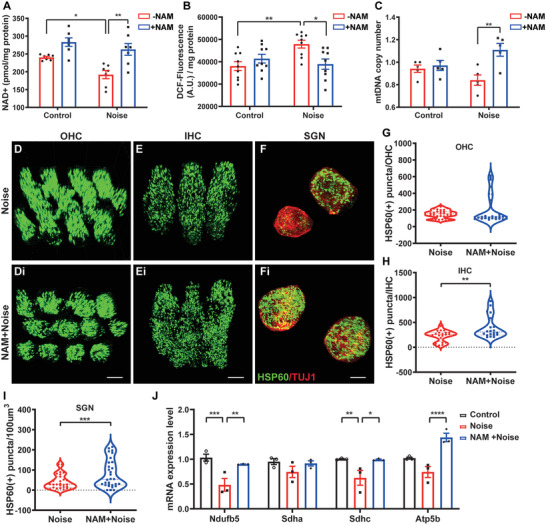
NAM maintained mitochondrial homeostasis from noise exposure in vivo. A) NAD^+^ level normalized by protein level in cochleae obtained from mice with/without noise exposure and NAM delivery (*n* = 6 cochleae). B) ROS level measured by DCFH‐A staining and normalized by protein level in the cochleae of mice with/without NAM delivery 4 h after noise exposure (*n* = 10 cochleae). C) mtDNA copy number in the cochleae of mice with/without NAM delivery at 3 dpn (*n* = 5 cochleae). D–F) 3D reconstruction immunofluorescence image by Imaris in the cochleae of mice with/without NAM delivery at 3 dpn, stained with HSP60 (Green) and TUJ1 (Red). Scale Bar: 5 µm. G–I) Bar graphs represented the numbers of HSP60 puncta in OHCs, IHCs and SGNs (*n* = 30 cells from 4 mice). J) mRNA levels of the genes relative to OXPHOS in the cochleae of mice with/without NAM at 1 dpn (*n* = 3 mice). Data were analyzed by t test, one‐way or two‐way ANOVA and represented as mean ± SEM. *P < 0.05, **P < 0.005, ***P < 0.0005, ****P < 0.0001.

**Figure 5:**
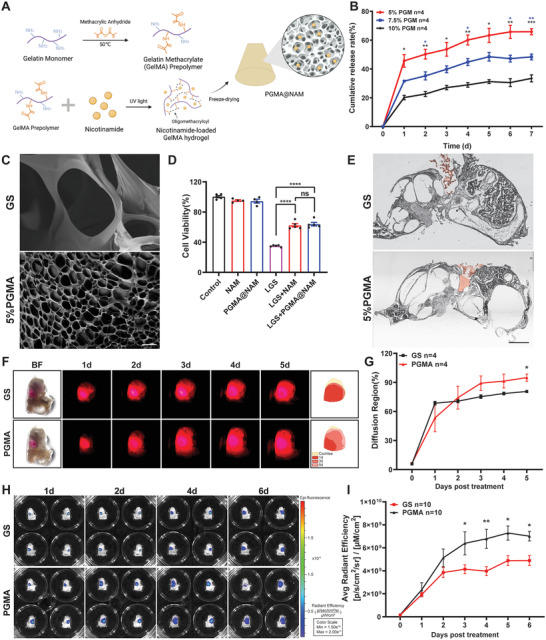
PGMA@NAM sustained release NAM in vitro. A) Scheme for PGMA@NAM production by gelatin methacrylation, cross‐linking and freeze drying. B) The cumulative release of Alizarin red S was evaluating by incubating PGMA in ddH2O at 37 °C and measuring absorbance at 424 nm (*n* = 4). C) Representative scanning electron microscope images of GS and PGMA. Scale bar: 20 µm. D) Cell viability of Neuro‐2a treated in 120 mm LGS with PGMA@NAM leaching liquid or 500 um NAM for 24 h (*n* = 4). E) Representative image of frozen section of the cochleae at 1 day after NAM delivery by PGMA or GS, highlight area represents GS/PGMA. F,G) Series images of GS and PGMA loading Rhodamine B diffused in the cochleae, the diffusion areas in cochleae were measured at different time (*n* = 4). Scale bar: 500 µm. H,I) Noninvasive bioluminescent imaging of rhodamine B fluorescence of the cochleae at 1, 2, 4, and 6 days after agent delivery with GS or PGMA, and the average fluorescence intensities in the whole cochleae were calculated (*n* = 10). Data were analyzed by one‐way or two‐way ANOVA and represented as mean ± SEM. *P < 0.05, **P < 0.005, ***P < 0.0005, ****P < 0.0001.

As we verified GS‐delivered NAM prevents noise‐induced auditory dysfunction, we next examined whether the protective efficacy of NAM treatment from noise‐damage–inducing conditions was by maintaining mitochondrial function. It is shown that NAM‐pretreated ears displayed approximately a 36.8% ± 10.5% increase in NAD^+^ levels and a 20.8% ± 7.1% decrease in ROS levels compared with the contralateral (non‐treated) ear following noise exposure (**Figure** **4**A,B). NAM treatment also robustly increased mtDNA copy number (by 32.1% ± 22.1%) in noise‐exposed cochleae (Figure 4C), indicating an increase in mitochondrial number. To further verify the involved target cell type(s), we labeled mitochondria by staining for heat shock protein 60 (HSP60) and counted individual OHCs, IHCs, and SGNs using a 3D reconstruction (Figure 4D–F). The number of mitochondria in OHCs did not differ between NAM‐treated and untreated cochleae at 3 dpn (Figure 4G), whereas the numbers of mitochondria in IHCs and SGNs (TUJ1‐labeled) of NAM‐treated cochleae were increased by 1.7‐ and 2.1‐fold, respectively, relative to those in untreated cochleae (Figure 4H,I). TEM‐based quantification further confirmed that the number of mitochondria in SGNs was increased by ≈18 per 100 µm^2^ (98.5 vs 80.8) in NAM‐treated cochleae (Figure S6A–D, Supporting Information), indicating that NAM protects cochlear cell mitochondria against noise trauma. Additionally, OXPHOS‐related gene expression was substantially upregulated by NAM treatment (Figure 4J), demonstrating that NAM enhances mitochondrial function and suppresses the ROS accumulation triggered by acoustic trauma in vivo. These results indicate that NAM promotes mitochondrial function in noise‐exposed cochleae in vivo through maintenance of mitochondrial dynamics. NAM protected NIHL in mice through RW delivery by GS two days before noise, but we found no protective effect in the noise‐exposed group after 5 days of NAM treatment (Figure S5F, Supporting Information), consistent with the results of a previous study.^[^ ^42^ ^]^ Histological examination revealed limited dispersion capacity in the RW niche (Figure 5E), providing an explanation for the inability of GS‐delivered NAM to provide long‐term protection against NIHL.

### 2.5 PGMA‐Mediated Sustained Release of NAM In Vitro

To achieve long‐term release in the cochlea from the RWM, we constructed NAM‐loaded porous gelatin methacryloyl (PGMA@NAM) by gelatin methacrylation and freeze drying (**Figure** **5**A). Through freeze‐drying, the solid hydrogel was able to achieve a micropore‐containing structure that allowed air conduction of sound and improved the capacity of gelatin to release drug and adhere to the RWM compared with GS and GelMA (Figure 5B; Figure S7A–D, Supporting Information). To visualize and quantify drug release in vitro, we generated PGMA loaded with different wt.% (5%, 7.5% and 10%) of the red dye, Alizarin red S, mimicking a small‐molecule drug. During the drug‐release test in vitro, Alizarin red S release slowly increased over 7 days. As shown in Figure 5B and Figure S7E (Supporting Information), the 5% group showed a higher release rate throughout the process than groups with higher concentrations of GelMA; thus, we selected 5% PGMA for subsequent experiments. A scanning electron microscopy (SEM) analysis of the surface morphologies of GS and PGMA showed that PGMA exhibited a microporous network with much smaller pore sizes compared with GS (Figure 5C; Figure S7F, Supporting Information). To evaluate the biocompatibility of PGMA@NAM and its capacity to protect against neural excitotoxicity, we co‐cultured Neuro‐2a cells with PGMA@NAM leachate (collected on day 2). We found that PGMA@NAM leachate did not affect cell viability, demonstrating the biocompatibility of PGMA@NAM (Figure 5D). It also exerted a protective effect similar to that produced by NAM treatment, increasing cell viability from 33.1% to 62.9% following LGS‐induced neural excitotoxicity, demonstrating that PGMA@NAM rapidly released NAM in vitro (Figure 5D). Cochlea are located in the inner side of the middle ear and are connected to the posterior cranial fossa by the internal auditory meatus (IAM), which is filled with lymphatic fluid. To illustrate the sustained release of small‐molecule drugs in the cochlea, we established a system for visualizing drug‐mimetic dispersion in the inner ear. To this end, we harvested cochleae from adult mice, then pinned them to a silicone elastomer‐coated dish with the RW exposed to air and IAM submerged in silicone, mimicking the cochlear‐adjacent structure in temporal bone (Figure 5E,G). To compare small‐molecule drug diffusion from the RWM, we selected rhodamine B for encapsulation in GS or PGMA. As shown in Figure 5E,F, fluorescence intensity continuously increased and diffused throughout the cochlea in the PGMA group at day 5, surpassing the effects observed in GS groups. We further measured the release of rhodamine B, based on its fluorescence intensity, using an in vivo imaging system (IVIS) (Figure 5G,H). GS showed an initial burst release at day 2 and then reached a plateau, whereas fluorescence intensity gradually increased in the PGMA group, peaking on day 5. PGMA fluorescence was the strongest from day 4 to 6, demonstrating the effective sustained capacity of PGMA ex vivo. These results show that 5% PGMA forms a microgel with a smaller pore size, allowing it to release drugs more effectively over a larger diffusion region and with a longer sustained duration compared with traditional GS.

### 2.6 Personalized PGMA@NAM Provides Long‐Lasting Protection from NIHL

**Figure 6:**
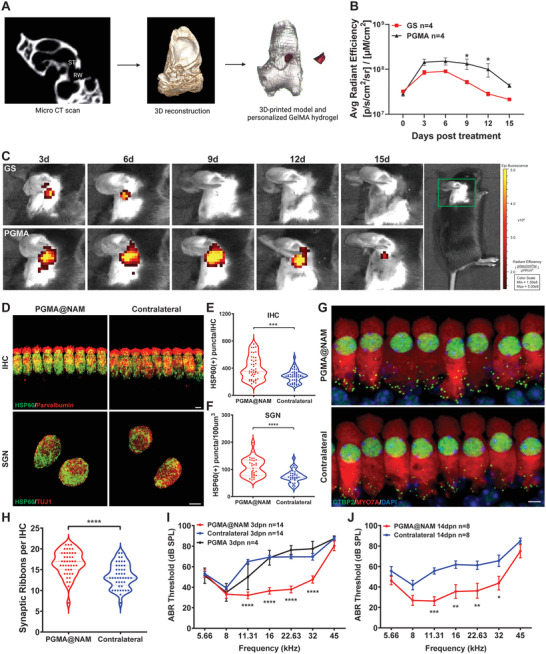
PGMA@NAM provided long‐lasting protection from NIHL. A) Scheme of manufacturing personalized 3D mold for PGMA@NAM production by micro CT scanning, 3D reconstruction and DLP printing. B,C) Noninvasive bioluminescent imaging of rhodamine B fluorescence to mice at 0, 3, 6, 9, 12, and 15 days after agent delivery with GS or PGMA, and average fluorescence intensities in the cochleae region were quantified (*n* = 4 mice). D) 3D reconstruction immunofluorescence image by Imaris in the cochleae of mice with/without PGMA@NAM delivery at 3 dpn, stained with HSP60 (Green) and Parvalbumin(Red) in IHC and TUJ1 (Red) in SGN. Scale Bar: 5 µm. E,F) Bar graphs represented the numbers of HSP60 puncta in IHCs and SGNs (*n* = 31 cells from 4 mice). G) Representative confocal images stained with Myo7a (Red) and CTBP2 (Green) in the IHCs at 16 kHz of the cochleae at 14 dpn. Scale Bar: 10 µm. H) Bar graphs represented the numbers of synaptic ribbons counting by ctbp2 puncta in each IHC (*n* = 18 IHCs from 3 mice). I,J) ABR threshold of all frequencies of the mice with/without PGMA or PGMA@NAM delivery at 3 and 14 dpn (*n* = 4–14 mice). Data were analyzed by t test or two‐way ANOVA and represented as mean ± SEM. *P < 0.05, **P < 0.005, ***P < 0.0005, ****P < 0.0001.

The topography of the RW niche—an irregular bony pouch of the tympanic cavity—has not yet been described in the mouse. To produce personalized NAM‐loaded GelMA which could achieve hydrogel in situ placing on RW niche, micro‐CT (computed tomography) was conducted to cochleae, finding similar shape and size of RW niche in different cochleae. we calculated the dimensions of the RW niche by micro‐CT, which yielded a diameter of ≈0.37 mm (Figure S8A, Supporting Information). PGMA is proved as a sustained release material for NAM in mouse, next we sought to implant sufficient PGMA in RW niche for long‐term release. Acrylic resin digital Light Processing (DLP) 3D‐printing was conducted to manufacturing 3D mold of cochleae based on micro‐CT 3D reconstruction, which was subsequently applied to GelMA in situ production (**Figure** **6**A; Figure S8B, Supporting Information), following conversion to PGMA. The fit of this personalized PGMA in the RW niche was tested by observing the spatial location of PGMA in temporal bone by micro‐CT. Iomeprol‐loaded PGMA was then placed into the RW niche of mice by postauricular bullostomy, and the cochlea was monitored by micro‐CT 1 day after surgery. Radiography results showed that the porous material was located in the RW niche and was positioned a subtle distance from the RWM (Figure S8C,D, Supporting Information), indicating that PGMA should not affect RWM vibration. To compare drug release in the inner ear, we established visual‐based analysis using IVIS detection of rhodamine B, allowing us to observe drug release speed and intensity daily. GS and PGMA encapsulated with rhodamine B were implanted in the RW niche, followed by IVIS testing to determine whether drug was immobilized and continuously released. We observed a distinct fluorescence signal in the injected ear that increased in intensity in the GS group from day 6 and ultimately disappeared on day 12, whereas no fluorescence decrease was detected throughout the 12‐day period in the PGMA group (Figure 6B,C), supporting the long‐lasting release of a small‐molecule drug by PGMA in inner ear. We next explored the long‐lasting protective effect of PGMA@NAM against NIHL in mice. PGMA@NAM or PGMA without NAM were placed on the RWM of mice 5 days before noise exposure. At 3 dpn, mitochondria (HSP60‐labeled) in IHCs and SGNs (parvalbumin‐labeled) were observed and counted after 3D reconstruction. As shown in Figure 6D–F, HSP60‐positive puncta in IHCs and SGNs from cochleae of the PGMA@NAM group were increased by 1.42‐ and 1.5‐fold, respectively, compared with untreated cochlea, indicating the long‐lasting effects of PGMA@NAM on mitochondria. A further analysis of synapses in the inner ear showed an increase in synapses (3 per IHC at 16 kHz) in PGMA@NAM‐treated mice at 14 dpn (Figure 6G,H). We also measured ABR thresholds in PGMA@NAM and NAM‐loaded gel‐foam groups at 3 and 14 dpn. This analysis showed that, although ABR thresholds did not differ between PGMA‐treated and contralateral groups (Figure 6I), PGMA@NAM treatment significantly ameliorated hearing loss compared with contralateral and PGMA groups at 3 and 14 dpn (Figure 6I,J), exhibiting 32.9, 32,9, 21.8, and 22.14 dB ABR threshold improvements at 11, 16, 22, and 32 kHz, respectively, at 3 dpn (Figure 6I). Notably, the protective effect of PGMA@NAM was maintained until 14 dpn (Figure 6J).

Collectively, our findings demonstrate the generation of a personalized PGMA designed to fit into the irregular murine RW niche without interfering with sound transmission. Moreover, PGMA@NAM exhibited sustained drug release by virtue of its porous structure and promoted hearing preservation by dramatically prolonging the function of NAM in vivo, thereby protecting against NIHL.

## 3 Discussion

In this study, we showed that noise exposure resulted in mitochondrial dysfunction and NAD^+^ depletion in murine cochleae. Pharmacologic augmentation of NAD^+^ through application of NAM rescued mitochondrial homeostasis, thereby protecting relevant models from neuroexcitatory toxicity in vitro and in vivo. We showed that NAM preserved synapses in IHCs and increased mitochondrial numbers in SGNs and IHCs after noise exposure. We demonstrated that pre‐treatment with NAM ameliorated auditory impairment after noise. Given the failure of traditional GS delivery to achieve long‐term drug release in the cochlea, we developed personalized NAM‐encapsulated PGMA as an otic drug‐delivery vehicle for NIHL, ensuring efficacy without interfering with sound transmission. PGMA@NAM exhibited long‐term release of NAM in the cochlea ex vivo and in vivo, providing a larger diffusion region in cochleae and extended protective efficacy against mitochondrial and synaptic damage and hearing loss after noise.

### 3.1 Supplement of NAD^+^ Precursor, NAM, is an Effective Therapeutic for NIHL

There is no effective therapeutics medicine in clinical for NIHL, and the main reason is the unclear status of cochlear damage after noise trauma. The mechanism underlying noise‐induced cochlear damage is still unclear, and there is debate regarding which cell type(s) experiences the initial damage. Noise can cause a PTS through permanent injury or loss of HCs, supporting cells, and neurons.^[^ ^43^ ^]^ Noise trauma might initially target a vulnerable subset of neurons, a concept supported by the finding that ≈50% of synapses are lost in the absence of HC loss in response to a temporary threshold shift (TTS) produced by noise exposure,^[^ ^44^ ^]^ which is thought to induced by glutamate dependent neural excitotoxicity.^[^ ^45^ ^]^ In our study, we found that exposure of mice to 104 dB SPL noise for 2 h resulted in permanent deafness in rodents, but without significant pathological changes in HCs or nerve fibers at 3 dpn. However, a TEM analysis revealed distinct alterations in mitochondrial morphology in SGNs and IHCs after noise exposure. Initially damaged cell types in cochleae after noise perhaps depends on intensity of noise: partial loss of mitochondria and swelling of mitochondrial membranes was reported in OHCs of rats that received 120 dB noise exposure for 5 h,^[^ ^46^ ^]^ whereas mitochondria in SGNs showed significant morphological disruption in rats or mice exposed to 115 dB noise for 3–6 h.^[^ ^47^ ^]^ Here, we identified SGNs and IHCs as priority targets for NIHL treatment, so we can screen drugs in neuronal cell line with LGS‐induced excitotoxicity insult, mimicking noise‐induced neural excitotoxicity.

To explore mechanism of NIHL, we conducted RNAseq of cochlea 1 day after noise trauma and found NAD^+^ metabolism and mitochondrial dysfunction is initially affected. NAD^+^, the cell's hydrogen carrier for redox enzymes, is well known for its role in redox reactions, genomic stability, gene expression, RNA processing, energy metabolism, immunity, inflammation, and the circadian clock.^[^ ^48^ ^]^ Here, we report that NAD^+^ is significantly depleted in cochleae after noise exposure, indicating that boosting NAD^+^ could be a promising option for NIHL treatment. Among NAD^+^ precursors that might be utilized for such an NAD^+^‐boosting strategy (i.e., NR, NMN, NA and NAM), NR has been shown to prevent NIHL and ARHL.^[^ ^18^, ^28^ ^]^ However, its short stability in the circulation and NRK‐expression–limited utilization has restricted its clinical application.^[^ ^48^, ^49^ ^]^ NMN has been shown to exert beneficial effects on age‐ and diet‐induced diabetes, acute heart failure, renal injury and neurodegeneration; it also protects against cisplatin‐induced ototoxicity.^[^ ^27^, ^50^ ^]^ NMN appears to have good bioavailability, given its rapid absorption and conversion to NAD^+^ in various organs (e.g., skeletal muscle, kidney and live), but how it is transported into cells remains unclear.^[^ ^48^ ^]^ The clinical application of NA has been limited because it induces cutaneous flushing at pharmacologically effective doses.^[^ ^51^ ^]^ Completing this list is NAM, an uncharged molecule that can diffuse rapidly across the plasma to support NAD^+^ biosynthesis in most tissues in vivo.^[^ ^52^ ^]^ Given that NR was previously assessed in the context of NIHL, we herein compared the other three abovementioned NAD^+^ boosters for their potential as NIHL therapeutics. In vitro, NA treatment was less effective than NAM or NMN in protecting neuronal cells against excitotoxic damage, a finding consistent with a report that the NAD^+^‐boosting capability of NAM is higher than that of NA in different organs of mice.^[^ ^48^ ^]^ Although LGS treatment to Neuro‐2a cells could not completely mimic mitochondria dysfunction on the mice cochleae after noise trauma, as LGS did not directly influence OXPHOS or respiratory chain in mitochondrial ROS excess production has been reported in mice cochleae after noise exposure, raising rapidly and decreasing over time,^[^ ^53^ ^]^ we can observe that LGS induced excitatory toxicity to Neuro‐2a cells, causing cell death as well as ROS production, which could be alleviated by NAD precursor^[^ ^39^ ^]^ (Figure 2B,D,E). Because the protective efficacy of NAM was less variable than that of NMN, we chose NAM for subsequent experiments designed to test protective effects of NAD^+^ precursors against NIHL. Indeed, NAM exhibited promising therapeutic effects against NIHL in vivo upon local administration, suggesting its viability for treating NIHL in the clinic. Taken together, our study and previous studies show that NR, NMN, and NAM are all potentially effective therapeutics for treating deafness,^[^ ^18^, ^28^ ^]^ reinforcing our view that NAD^+^ argumentation is a sound strategy for prevention of deafness.

Nevertheless, the mechanism of NAM protection from NIHL has not been illustrated in our study. It was proved that NAM could provide neuroprotective effects under stress through preventing metabolic disruption, increasing mitochondrial size and motility, which was reported in studies of glaucoma in rodents.^[^ ^31^, ^54^ ^]^ Additionally, as a cofactor or co‐substrate for many enzymes and NAD^+^ supplementation by NR or NMN can promote injured mitochondria clearance via mitophage,^[^ ^55^ ^]^ facilitate DNA repair^[^ ^24a^ ^]^ and regulate lipid metabolism via PPARγ signaling as well,^[^ ^28b^ ^]^ which could illustrate potential mechanism of NAM protective effective from NIHL.

### 3.2 Personalized Round Window Delivery is an Effective Approaching for NIHL Treatment

The efficacy of systemic drug therapy is limited by restricted uptake into the inner ear by BLB, one of the physiological barriers that separate the inner ear from the external system. BLB provides a diffusion barrier that excludes many substances entering the inner ear from the blood, thus lowering the efficiency of drug delivery. Treatment for hearing loss such as NIHL could be administered systemically, while systematic drug administration often demonstrates limited bioavailability, with low local drug concentrations in the inner ear.^[^ ^34^ ^]^ And the potential protective agent may not cross the BLB toward the targeted cells in cochlea and may cause side effects in other organs.^[^ ^56^ ^]^ Therefore, local drug delivery strategies are more suitable and efficient for inner ear therapy. For local application, there are two minimally invasive approaches, injection by intra‐tympanic administration or application of drugs directly to the RWM, which locates at the bottom of the RW niche. Virus for gene replacement or gene editing reagents, which are hard to be infused from RWM, are always injected through intra‐cochlear.^[^ ^57^ ^]^ For small molecular drugs, application of drugs directly to the RWM is safe and efficient delivery approaching. Additionally, delivery from RWM is much easier for operation and more feasible for clinical translation.

Besides, the cavity of RW niche is an ideal space for drug storage, which is a potential advantage for long‐term drug release. To maximize the drug storage, we here reconstructed the RW niche cavity based on micro‐CT‐assisted topography and printed a custom‐fit delivery system via DLP 3D‐printing technology for it. This personalized delivery system was able to completely attach to the RWM, enabling maximum delivery efficiency into the inner ear. Scans of temporal bones from six adult mice revealed no distinct difference in RW niche size, even at different ages of audlt mice, showing that we can use the uniform delivery model for adult mice. Such personalized delivery systems could be optimized for different species by utilizing our strategy for designing personalized inner ear delivery systems based on micro‐CT‐assisted topography and 3D printing. Although the accuracy of DLP based 3D printing (≈1 mm) limits the direct production of PGMA@NAM with tiny size, it could be applied to mammals even humans with bigger size of RW niche. In addition to providing maximal RWM contact area and delivery capacity, this personalized delivery systems are also easy to operate, which makes it a promising strategy for future clinical translation.

### 3.3 Porous GelMA is an Effective Material for Long‐Term Sustained Drug Release in Inner Ear

To achieve sufficient contact with the RWM and realize the sustained release of the drug, a proper delivery material is required. GelMA is a versatile material for a wide range of bio‐applications, which has good biocompatibility and can be easily shaped and photo‐cross‐linked under UV light, making it a satisfying drug delivery vehicle. The methacryloyl group allow GelMA to form covalently cross‐linked hydrogel under visible light, achieving in‐situ gelation in short time.^[^ ^58^ ^]^ Comparing to other cross‐linking modes, including heat‐, pH‐, enzyme‐triggered cross‐linking approach, light‐curing strategy enables the hydrogel to be stable and easy to operate, thus making it an excellent choice for in vivo application. Additionally, thanks to the hydrophilic functional groups attached to the 3D network backbone, GelMA is able to hold water inside the hydrogel network, making it an excellent hydrophilic drug carrier.^[^ ^59^ ^]^ However, it is not widely used in deafness treatment^[^ ^35^, ^37^ ^]^ since the direct injection and photo‐cross‐link leave GelMA in bulk form without pores, which tends to impede the vibration of RWM and interfere with sound conduction.^[^ ^60^ ^]^ Utilizing microfluidic technology to generate GelMA microgels can avoid this problem because they are microparticles with interspace, allowing sound transmission.^[^ ^37^ ^]^ Nevertheless, their point contacts with the RWM have limited contact area, thus lowering their therapeutic effects. To generate a vehicle that has enough contact area with RWM without interfering with sound conduction, we develop porous GelMA via freeze drying technique to meet these needs. Here, we found that porous GelMA is safe and efficient for drug delivery, with satisfying sustained release capacity. Although we have not examined the longer lasting protect effect of PGMA@NAM from NIHL, it was observed that PGMA possessed 12‐day drug release, suggesting potential of PGMA@NAM prospectively long‐lasting protective efficacy with slow‐degrading characteristics.^[^ ^61^ ^]^ Porous GelMA achieved sufficient long‐term release for NAM in inner ear. Diffusion behavior of small molecules (nutrients and waste) in cell culture and drug delivery system from GelMA and hydrogel degradation might depend on humidity.^[^ ^61^, ^62^ ^]^ It is known that tympanic cavity is an air‐filled compartment surrounded by bones, with communication with pharynx via eustachian tube that help drain fluid and equalize atmosphere in middle ear. Thus, making balance among water retention, drug sustained‐release and sound conduction in RW niche is worth considering, for example, electrospun hydrogel,^[^ ^63^ ^]^ double‐hydrophobic coating hydrogel,^[^ ^64^ ^]^ or thiolated chitosan (TCS) photocross‐linked GelMA.^[^ ^65^ ^]^ Collectively, our findings demonstrate that PGMA is an excellent drug‐delivery platform for the treatment of hearing loss, and we are exploring more effective and long‐term release delivery material for NIHL protection.

In this study, we established custom‐fitted NAM@PGMA—a personalized PGMA delivery system that can achieve long‐term hearing protection against NIHL—providing a promising therapeutic option for treating deafness in the clinic.

## 4 Experimental Section

### Animal Procedures

All studies involving animals were bred in qualified facilities (SYXK2020‐0025) and animal procedures (SH9H‐2020‐A102‐1) were approved by the Ethics Committee of the Shanghai Jiaotong University school of medicine. C57BL/6J male mice were purchased from Shanghai Jihui Laboratory Animal Care Company Limited. Animals were randomly assigned to each group with at least three mice.

### NAM Supplementation on Mice

Four‐week‐old C57BL/6J male mice were used for NAM local supplementation via bullostomy. The mice were anesthetized using 50 mg kg^−1^ Zoletil50 (WK001, Virbac S.A.) and 20 mg kg^−1^ xylazine (X1251, Sigma Aldrich‐Fluka, St. Louis, MO, USA) delivered through intraperitoneal injection. The right postauricular region of the mice was shaved to expose and sterilized with 10% povidone iodine. The right bullas of the mice were exposed and opened to access the round window membrane with drilling by a fine needle, onto which absorbable gelatin sponge (GS) with size of 0.3 × 0.4 × 0.5 mm saturated with nicotinamide (20 mg ml^−1^) or PGMA@NAM were placed, ≈1 µg NAM was placed into RW niche. Then the bullostomy was sealed by tissue adhesive (3 m Vetbond, St. Paul, MN) and the skin incisions were closed, and 2 days or 5 days after supplementation the mice would receive noise exposure.

### Noise Exposure

The mice were awake, unrestrained and exposed to an 8–16 kHz octave‐band noise for 2 h at 104 dB SPL, and then placed in an acoustically transparent wire‐netting cage. The noise signal was generated by TDT System3 RZ6 Processor (Tucker DAVIS Technologies, Alachua, FL USA), filtered, amplified by a Crown power amplifier, and delivered to a loudspeaker fix securely to the hole in the top of the cage. Sound levels were adjusted with a condenser microphone (PCB PIEZOTRONICS, 480C02), which within the cage vary by <1 dB SPL.

### Auditory Brainstem Response Testing

ABR tests were recorded using the TDT System3 RZ6 workstation (Tucker Davis Technology, Alachua, FL USA). Mice were anesthetized by 50 mg kg^−1^ Zoletil50 and 20 mg kg^−1^ xylazine through intraperitoneal injection. Body temperature was maintained at 37 °C throughout recording with a Homeothermic Monitoring System (Harvard Apparatus, 55–7020). Three needle electrodes were inserted into the subcutaneous layer of the skin, including vertex (active electrode), mastoid region (reference electrode) and the dorsal skin of buttock (ground electrode). Stimulus sounds were presented close field with the decreased in 5 dB SPL steps from 90 dB SPL at the stimulus frequencies of 5.66 to 45 kHz. Thresholds were defined manually as the lowest dB SPL level at which any wave could be detected.^[^ ^66^ ^]^

### Distortion Product Otoacoustic Emission Testing

DPOAE were recorded from anesthetized mice. The sound stimulus consisted of simultaneous permanent pure tones at two different frequencies (f2/f1 ratio = 1.22) decreasing from 80 to 10 dB (L1 = L2) in 5 dB steps. Distortion product otoacoustic emissions (DPOAEs) were measured at five frequencies from 5.66 to 32 kHz. The DPOAE signals were displayed using TDT software. The hearing thresholds for each frequency were defined as a numerical value of the last detectable amplitude that was produced in response to the acoustic stimulation.

### Cochlear Explants

P2 C57BL/6J mice cochleae were disserted in HBSS supplemented with 1x HEPES and placed in 10 mm slides positioned in 35 mm dish with 4 rings, which had already been coated with Matrigel Matrix (#354 248, Corning). Cochlear explants were maintained in DMEM/F12 containing 1x N2 (A1370701, Sigma) and 1x B27 (17 504 044, Sigma) overnight at 37 °C with 5% CO2. Then they were randomly divided into groups, treated with/ without 0.5 mm NMDA (0114/50, Tocris) and 0.5 mm kainic acid (K0250, Sigma), co‐treated with/without test compounds. NK was removed 2 h later, remaining test compounds incubated for 18 h. Next the cochleae were fixed in 4% paraformaldehyde at room temperature for 30 min, followed by immunofluorescent staining.

### Immunohistochemistry

For whole‐mount immunofluorescence, animals were sacrificed by cervical dislocation. Inner ears were fixed in 4% paraformaldehyde (PFA) at 4 °C overnight, then decalcified in 0.5 m EDTA for at least 5 hrs. The cochleae were dissected in pieces or incubated in 15% and 30% glucose, followed by O.C.T embedding and frozen section, then blocked with 0.3% Triton X‐100 and 8% donkey serum for 1 h at room temperature before primary antibodies. They were labeled with rabbit anti‐Myosin VIIa (1:300, #25‐6790, Proteus BioSciences Inc), mouse (IgG1) anti‐Ctbp2 (1:300, #612 044, BD Biosciences) and anti‐NF‐H (1:500, #AB5539, Millipore) or rabbit anti‐TUJ1 (1:300, 801 202, Biolegend) overnight at 4 °C. For the pieces that should be labelled with anti‐HSP60 (1:200, #12 165, CST), antigen retrieval was required by incubating with citrate acid (10 mm, pH6.8) in 95 °C for 30 min. And 1:500 secondary antibodies followed for 1 h after three rinses with PBS. All Alexa Fluor secondary antibodies were from Invitrogen: Donkey anti‐rabbit Alexa Fluor 488 (A‐21206), goat anti‐mouse (IgG1) Alexa Fluor 488 (A‐21121), goat anti‐chicken Alexa Fluor 647 (A‐21449) and donkey anti‐rabbit Alexa Fluor 594 (A‐21207). DAPI (D3571, Invitrogen) was added 5 mg ml^−1^ in secondary antibodies at 1:1000 dilution to stain nuclei. After three rinses with PBS, specimens were mounted in ProLong Gold Antifade Mountant medium (P36934, Invitrogen). Confocal images were taken with a Zeiss LSM 880 fluorescence confocal microscope using a 63× or 100x glycerin‐immersion lens.

Ctbp2 puncta per IHC and number of mitochondria were measured in 3–4 different regions of every turn of the cochleae. Ctbp2 count, the number of mitochondria and 3D‐reconstruction were accomplished using Imaris software (Bitplane AG).

### Transmission Electron Microscopy

Cochleae were quickly removed from anesthetized and sacrificed mice, and perfused with 4% paraformaldehyde and 0.5% glutaraldehyde in ice. After fixed in 2.5% glutaraldehyde at 4 °C overnight, the cochleae were decalcified in 0.5 m EDTA for 5 h at 4 °C, followed by post‐fixed with 1% osmium tetroxide for 2 h at room temperature. For Neuro‐2a, same volume of 5% glutaraldehyde was added to the cell suspension, and fixed at room temperature for 20 min, then followed by centrifugation at 10 000 g for 5 min. Supernatant was removed and replaced by fresh 2.5% glutaraldehyde and fixed in room temperature for 1 h with post‐fixation for 2 h at 4°C. Then dehydration was required in a series of graded alcohols and acetone, and the samples were infiltrated and embedded in Epon 812 resin, then polymerization at 65°C for 48 h. The cochleae were cut at the level of organ of Corti in thin section, and the sectioned samples were double stained with uranyl acetate followed by lead citrate and examined with a 120 kV transmission electron microscope (FEI Talos L120C).

### NAD^+^ Quantification

NAD^+^ quantification was measured in cochleae tissue using NAD/NADH Quantitation Colorimetric Kit (#K337‐100, BioVision, USA), as per manufacturer's instructions. Briefly, the cochleae were taken and homogenized in 400 µl of NAD/NADH extraction buffer, respectively, then centrifuged at 14000 rpm for 5 min. Supernatants were divided for measurement of NADt, NADH, and protein content. NADH were determined by decomposing NAD^+^ at 60 °C, 30 min before performing detection reaction. Both NADt and NADH levels were measured at OD 450 nm. NAD^+^ levels were normalized to protein content determined by BCA assay (#23 225, Thermo Scientific, USA), and were determined by the following formula: (NADt‐NADH)/protein.

### Measurement of ROS Production

DCFH‐DA (D6883, Sigma) were used to ROS production measurement. Cochlear tissues were collected and homogenized in 300 µl 40 mm pH7.2 Tris‐HCl, then supernatants were divided for measure of DCFH‐DA and protein content. While cells were digested and made into cell suspension. For 2′,7′‐dichlorodihydrofluorescein diacetate staining, supernatants were incubated with DCFH‐DA (10 µM) for 30 min. The fluorescence was measured using microplate reader (Spark multimode microplate reader, Tecan, Switzerland) or flow chemistry (LSRFortessa, BD, USA).

### Quantification of mtDNA Copy Number

Total DNA was extracted from mouse cochleae without vestibule according to the manufacturer's instructions (TIANGEN, DP304‐03). And mtDNA copy number was determined with Real‐time PCR (Roche Lightcycler 480) by amplifying a portion of the *CoxI* gene of mtDNA and comparing it to the amplification profile of a nuclear single copy gene, *β‐actin* gene. Primer sequences are shown in Table S1 (Supporting Information).

### Quantitative Real‐Time Reverse Transcription Polymerase Chain Reaction (qRT‐PCR)

Total RNA was extracted from mouse cochleae without vestibule or the cultured cells with the optional DNase digest step according to the manufacturer's instructions (Qiagen, #74 136). The RNA was reverse transcribed with Evo M‐MLV RT Kit with gDNA Clean for qPCR (Accurate Biotechnology Co., Ltd., China) and amplified by qRT–PCR in Roche Lightcycler 480 with a SYBR Green Premix Pro Taq HS qPCR Kit (Accurate Biotechnology Co., Ltd., China). The PCR program was preincubation at 95 °C, 5 min, followed by 45 cycles of 95 °C, 10 s, 60 °C, 10 s, and 72 °C, 10 s. The relative levels of each target gene were normalized to endogenous Rpl19 and calculated using comparative Ct method (ΔΔCt method). Primer sequences are shown in Table S1 (Supporting Information).

### RNA Extraction, Library Construction and RNAseq

Total RNA was extracted from mouse cochleae without vestibule with the optional DNase digest step according to the manufacturer's instructions (Qiagen, #74 136). RNA purity was checked using the NanoPhotometer spectrophotometer (IMPLEN, CA, USA). RNA integrity was assessed using the RNA Nano 6000 Assay Kit of the Bioanalyzer 2100 system (Agilent Technologies, CA, USA). Short fragment libraries for RNA sequencing were made with poly‐A–selected mRNA using the Illumina TruSeq RNA library construction kits v2 (Illumina). The resulting libraries were purified using AMPure XP system (Beckman Coulter, Beverly, USA) and then were quantified using Agilent Bioanalyzer 2100 system. Libraries were pooled, requantified, and run as 150‐bp single‐end lanes on an Illumina Novaseq platform, using TruSeq PE Cluster Kit v3‐cBot‐HS (Illumia), and FASTQ files of paired‐end read files were generated.

### RNAseq Data analysis

RNAseq reads in the FASTQ files were first processed through in‐house perl scripts. Clean reads were obtained by removing reads containing adapter, reads containing ploy‐N and low‐quality reads from raw data. At the same time, Q20, Q30, and GC content the clean data were calculated. Genes and transcripts were annotated using featureCounts v1.5.0–p3. Genes with p‐values < 0.05 were marked as significant. To assess the extent of functional enrichment, GO enrichment analysis using clusterProfiler R package was performed, which determines the statistical enrichment of DEGs in GO pathways.

### Cell Culture

Neuro‐2a cells were grown in DMEM supplemented with 10% heat‐inactivated FBS, and 1% penicillin and streptomycin. SH‐SY5Y cells were grown in DMEM/F12 with 10% FBS, 1x Gluta‐max (Invitrogen, 35 050 061), 1x sodium pyruvate (Invitrogen, 111 360 070), 1% penicillin and streptomycin, maintained at 37 °C and 5% CO2. For western blot, qRT‐PCR, and flow cytometry, Neuro‐2a cells and SH‐SY5Y cells were grown in a 60 mm dish to 80%−90% confluence and treated with 120 mm L‐glutamate sodium and 0.5 mm NAM for at indicated time points before collected.

### Cell Viability Assessment

Neuro‐2a cells and SH‐SY5Y cells were plated in 96‐well plates (4 × 10^4^ cells per well). Cells were incubated with 100 µl drug‐supplemented medium in HBSS (1 g L^−1^ glucose). After incubation for 24 h, cell viability was measured using a Cell Counting Kit‐8 (CCK8) assay (Dojindo Molecular Technologies, Japan) according to the manufacturer's instructions. The optical density at 450 nm (OD450 nm) was measured using Tecan Spark multimode microplate reader.

### Flow Cytochemistry

Flow cytochemistry analysis of mitochondrial activity was performed on Neuro‐2a cells suspension. For mitochondrial activity, cells were incubated at 37 °C for 30 min with TMRM (Invitrogen, 134 361; 1:1000) or 10 um DCFH‐DA, and stained with DAPI (Invitrogen, D357; 1:1000). Twenty thousand cells were acquired and sorted by flow chemistry (LSRFortessa, BD, USA), then data was analyzed post‐acquisition using FlowJo software (Tree Star, Ashland, OR, USA).

### Preparation and Physical Characterization of PGMA

GelMA (EFL, China) was dissolved by following protocol. Water‐soluble photo initiator LAP was dissolved in PBS at 50 °C for 15 min, making a 0.25% (w/v) LAP standard solution. To obtain 10% (w/v) GELMA/LAP solution, GelMA was dissolved in filtered LAP standard solution at 65 °C for 20 min. For GelMA application, 10% (w/v) GELMA/LAP solution was mixed with the same volume of 40 mg ml^−1^ NAM, Rhodamine B, or Alizarin red S solution and injected into a transparent cochlear mold with RW niche, followed by cross‐linking under ultraviolet irradiated (405 nm). The NAM‐, Rhodamine B‐, and Alizarin red S‐loaded GelMA hydrogel were incubated overnight in a refrigerator at −80 °C and transferred to a vacuum freeze‐drier (Labconco, USA) for 3 days to obtain a porous structure. The porous GelMA hydrogel was stored at 4 °C for further use. Surface morphology of GS and PGMA were investigated by GeminiSEM 300 scanning electron microscope (SEM) (ZEISS, Germany) after sputter‐coating with gold.

### Cytocompatibility of PGMA

PGMA@NAM (30 ul) was incubated at 37 °C for 2 days in 3 ml HBSS with leachate collected every day. Neuro‐2a cells were co‐cultured with LGS and the leachate (collected on day 2) for 24 h and cell viability was measured.

### Release Profile of PGMA In Vitro

5‐, 7.5‐, and 10‐wt.% PGMA loading Alizarin red S were prepared as described above and incubated in 2 ml ddH2O at 37 °C for 7 days separately. Leaching liquid (0.2 ml) was collected at the predetermined time points with 0.2 ml fresh ddH2O added back. The concentration of Alizarin red S was measured at OD 424 nm to estimate the amount of Alizarin red S released from PGMA into ddH2O. To mimic drug dispersion in inner ear, cochleae were nailed in the dish with IAM submerged in silicone elastomer and RW exposed to the air. GS and PGMA encapsulating Rhodamine B were placed onto RWM, and fluorescence intensities were continuously observed by fluorescence stereo microscope and IVIS for 6 days.

### Micro‐CT Scanning of Cochlea

For analysis of RW niche of the cochleae, cochleae of mice were removed and fixed in 4% PFA. For analysis of PGMA location after delivery, PGMA loading Iomeprol was placed on RWM of mice by bullostomy, following with cochleae desertion a day after. Then the cochleae were scanned with micro‐CT 50 (Scanco Medical, Switzerland) in 10 µm voxel size. After scanning, all images were submitted to 2D images from different observation directions and 3D reconstruction of the microtissues. The image processing procedure were carried out under RadiAnt Dicom Viewer (Medicant, Poznan, Poland). Acrylic resin DLP‐based 3D printing was conducted based on the 3D reconstruction of the cochleae.

### Noninvasive Bioluminescent Imaging of Live Animals

After being anesthetized same as above and the furs of the head and back was shaved, mice were placed in the prone position of the imaging room. IVIS Lumina Series III (PerkinElmer) was used to perform noninvasive bioluminescent imaging of rhodamine B fluorescence in order to monitor NAM delivering efficiency in the PGMA.

### Statistical Analysis

Statistics were analyzed by GraphPad Prism v.8.0 program (GraphPad Software, San Diego, CA) and data was presented as the mean ± SEM. All experiments were repeated at least three time. One‐way ANOVAs with Bonferroni was used for analysis to mean difference and two sample Student's t test also applied if only two groups were compared. Two‐way ANOVA with Bonferroni was used for comparing the effects of more than one treatment as well as for functional hearing assessments. *P* values<0.05 were accepted as significant.

## Conflict of Interest

The authors declare no conflict of interest.

## Author Contributions

B.F., T.D., and X.S. contributed equally to this work. B.F., T.D., W.Z., Y.T., and H.W performed conceptualization. B.F., T.D., X.S., X.Z., W.Z., and Y.T. performed methodology, B.F., T.D., X.S., X.Z., and Y.T. performed experiment. B.F., T.D., X.S., X.Z., X.W., and Y.T. performed data analysis. X.W., Y.T., and H.W. performed supervision. B.F., T.D., X.S., and Y.T. wrote the manuscript. B.F., T.D., X.W., X.S., X.Z., C.J., Z.C., Y.L., W.Z., Y.T., and H.W. review and edited the manuscript.

## Supporting information

Supporting Information

## Data Availability

The data that support the findings of this study are available from the corresponding author upon reasonable request.
