# Supplementary material for: Personalized Porous Gelatin Methacryloyl Sustained‐Release Nicotinamide Protects Against Noise‐Induced Hearing Loss
Source: Adv Sci (Weinh). 2024 Jan 15;11(12):2305682. doi: 10.1002/advs.202305682 (PMC10966548; doi:10.1002/advs.202305682)
Supplement: Supplementary file 1 — Supporting Information [file ADVS-11-2305682-s001.pdf]

ADVANCED SCIENCE
Open Access

Supporting Information

for *Adv. Sci.*, DOI 10.1002/advs.202305682

Personalized Porous Gelatin Methacryloyl Sustained-Release Nicotinamide Protects Against Noise-Induced Hearing Loss

Baoyi Feng, Tingting Dong, Xinyu Song, Xiaofei Zheng, Chenxi Jin, Zhenzhe Cheng, Yiqing Liu, Wenjie Zhang, Xueling Wang, Yong Tao* and Hao Wu**

Supporting information

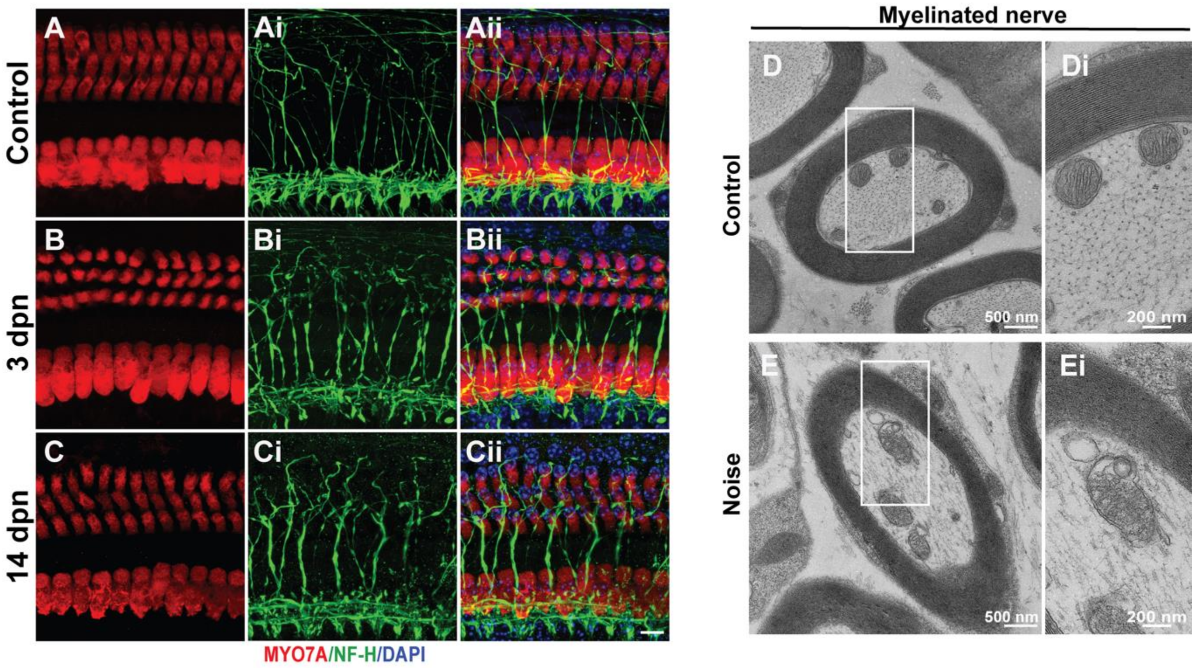

Figure S1 Cochleae and mitochondrial morphology in myelinated nerve after noise exposure (A-C) Representative confocal images of Myo7a (Red), NF-H (Green) at the 16kHz region of the cochleae in the mice before noise or at 3, 14 dpn. Scale bars: 10 μm. **(D and E)** Representative transmission electron microscopic images of mitochondria in myelinated nerve in the murine cochlea pre-noise and 1 dpn.

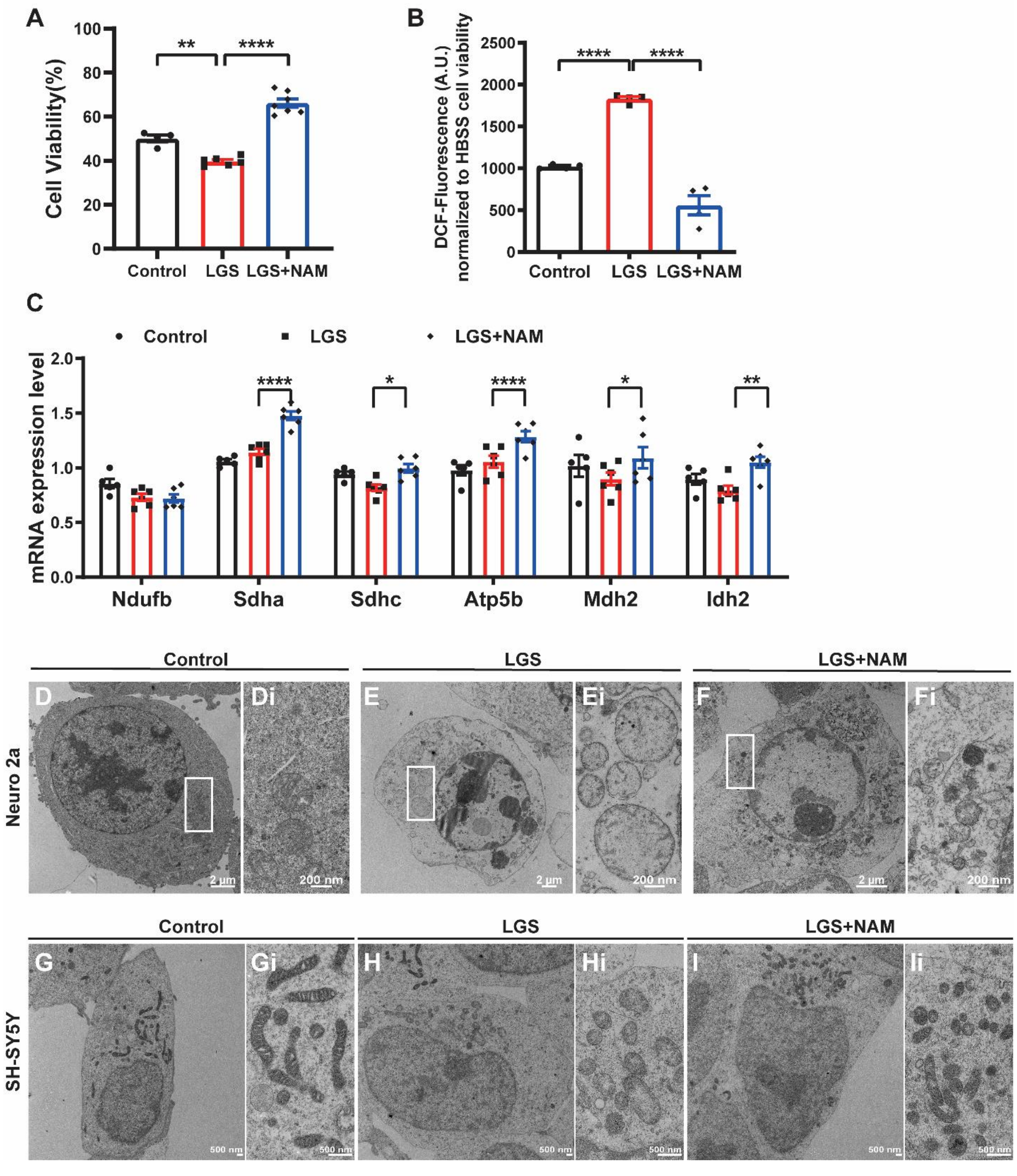

Figure S2 NAM protected Neuro-2a and SH-SY5Y from neural excitotoxicity

(A) Cell viability of SH-SY5Y treated in 120mM LGS with/without 500uM NAM (n=6). **(B)** ROS level measured by DCFH-A staining and normalized by cell viability of SH-SY5Y treated with LGS and NAM for 12 hours. (n=3). **(C)** mRNA levels of the genes relative to OXPHOS and TCA in SH-SY5Y treated in 120mM LGS with or without 500uM NAM for 12 hours (n=5). **(D and E)**

Representative TEM images Neuro-2a and SH-SY5Y cells treated with LGS and NAM. Mitochondria were emphasized in the images. Data were analyzed by one-way or two-way ANOVA and represented as mean ± SEM. *$P < 0.05$, **$P < 0.005$, ***$P < 0.0005$, ****$P < 0.0001$.

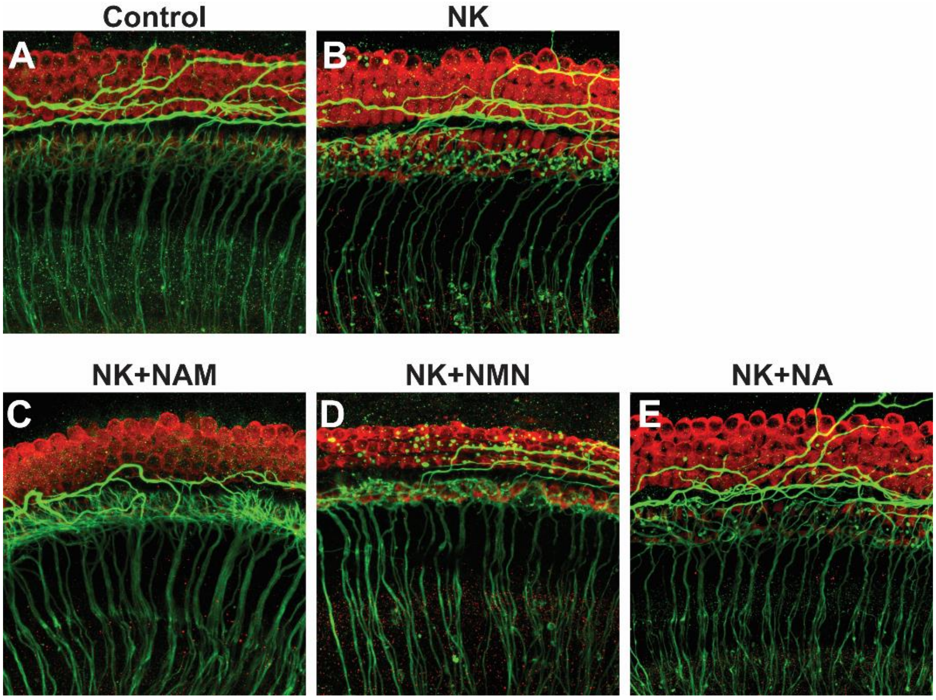

Figure S3 NAD$^+$ precursor protected cochlear explants from neural excitotoxicity

(A-E) Cochlear explants treated in NK with/without NAM, NMN and NA were stained with Myo7a (Red) to identify hair cells and NF-H (Green) to label neurofilament of SGN.

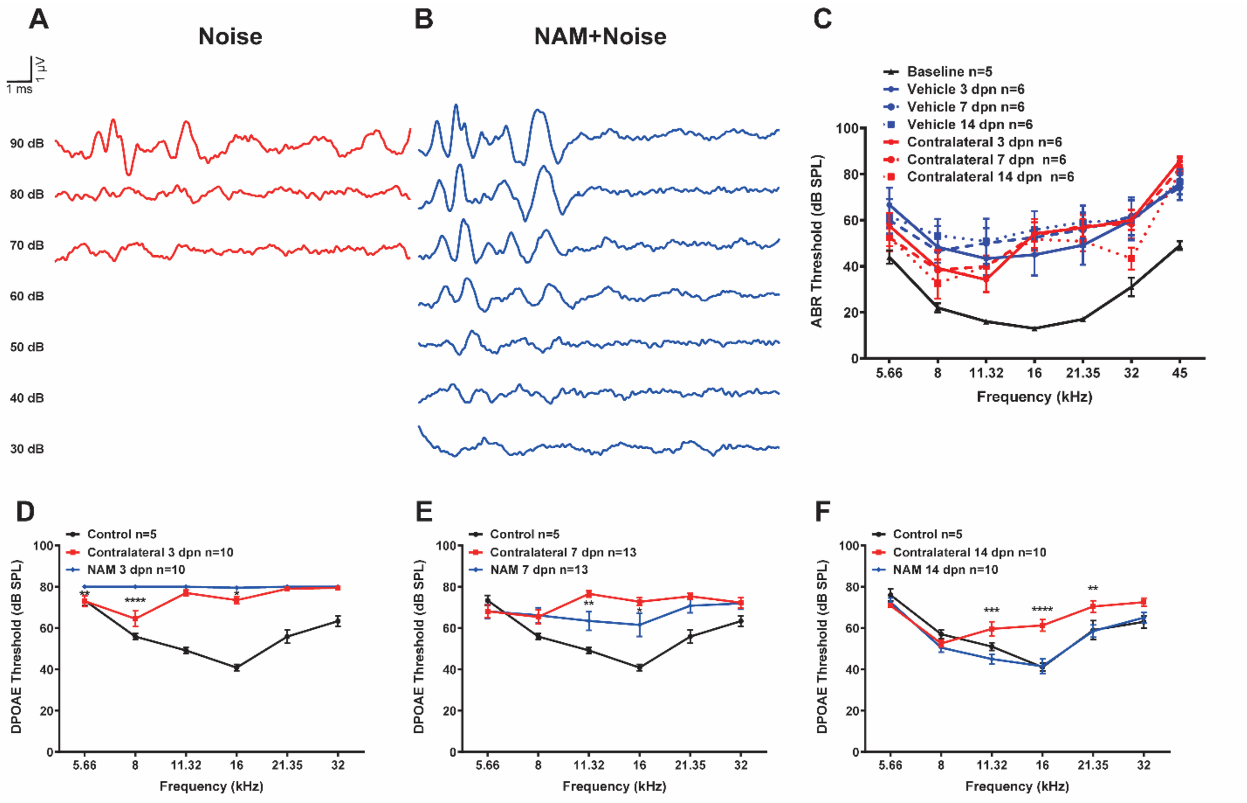

Figure S4 ABR waveforms and DPOAE of mice with NAM treatment

(A and B) Representative ABR waveforms recorded at 16 kHz with different stimulus intensity of the mice with/without NAM at 3 dpn. **(C)** ABR threshold of all frequencies of the mice with/without vehicle treatment at 3, 7 and 14 dpn (n=5-6 mice). **(D-F)** DPOAE threshold of all frequencies of the mice with/without NAM delivery at 3, 7 and 14 dpn (n=5-13 mice). Data were analyzed by two-way ANOVA and represented as mean ± SEM. **P* < 0.05, ***P* < 0.005, ****P* < 0.0005, *****P* < 0.0001.

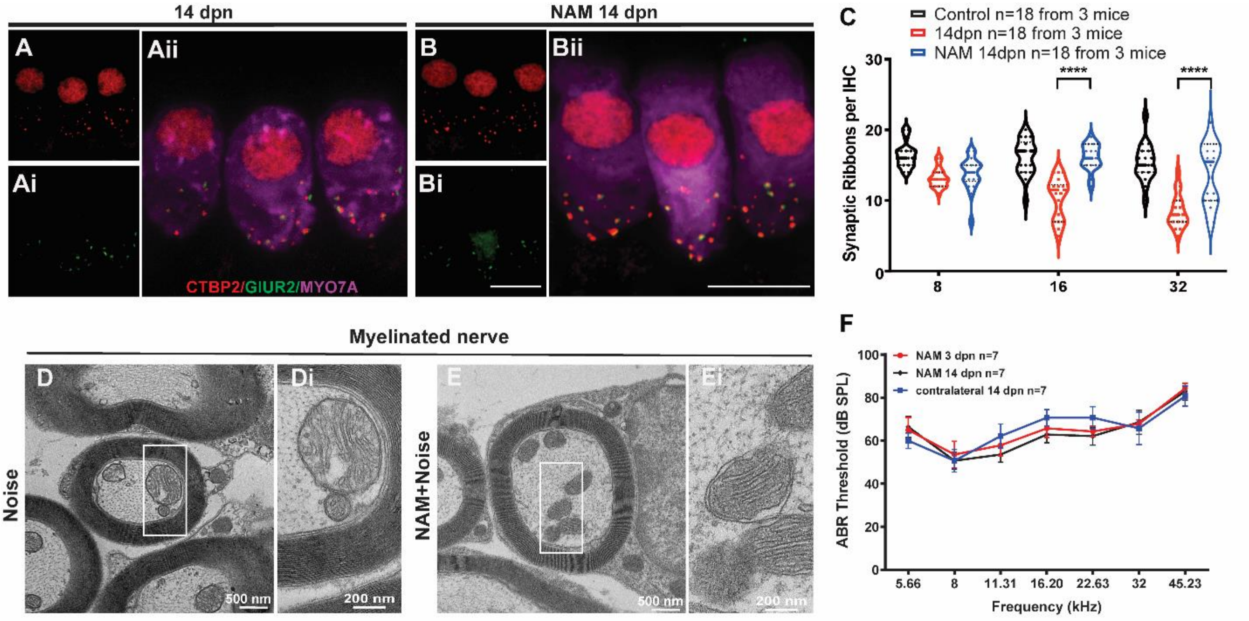

Figure S5 Synapses in cochlear IHC and mitochondria in myelinated nerve of mice with NAM treatment

(A-C) Confocal images and synaptic ribbons counting in each IHC at 14 dpn (n=18 IHCs from 3 mice). **(D and E)** Representative transmission electron microscopic images of mitochondria in myelinated nerve in the cochlea of the mice with/without NAM at 1 dpn. **(F)** ABR threshold of all frequencies of the mice at 3 and 14 dpn with/without GS@NAM delivery 5 days before noise exposure (n=7 mice). Data were analyzed by two-way ANOVA and represented as mean ± SEM. ****$P < 0.0001$.

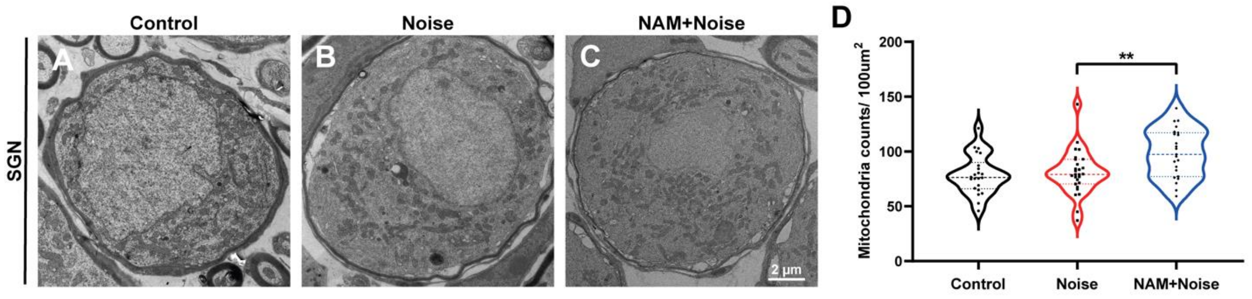

Figure S6 NAM maintained mitochondrial homeostasis in SGNs

(A-C) Representative transmission electron microscopic images of mitochondria in SGNs in the cochleae of the mice control and with/without NAM at 1 dpn. **(D)** Violin graphs represented the numbers of mitochondria in SGNs per um^2 counted under TEM (n=21 cells from 3 mice in each group). Data were analyzed by one-way ANOVA and represented as mean ± SEM. $^{**}P < 0.005$.

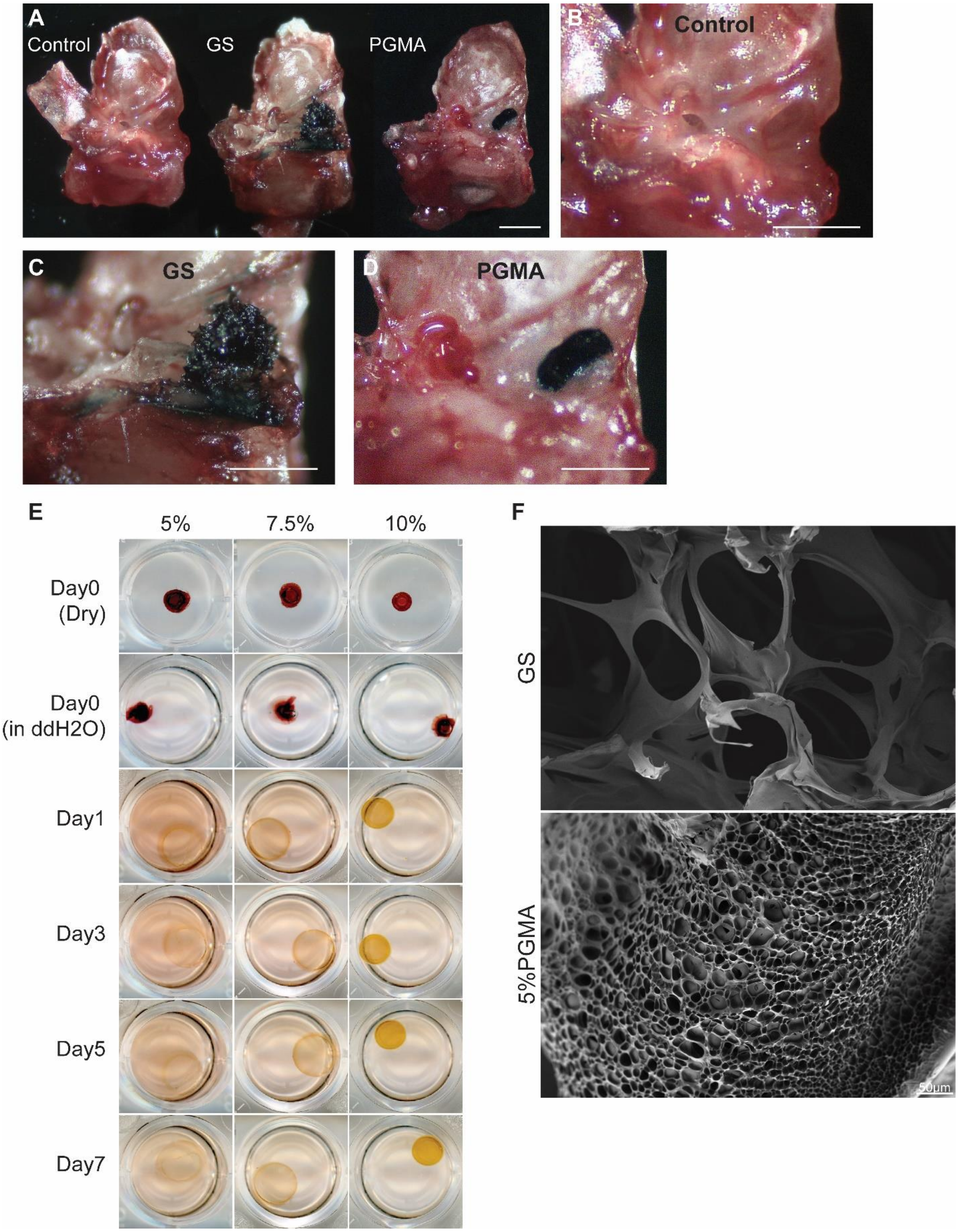

Figure S7 Drug release and surface morphology of PGMA

(A-D) Photograph under stereo microscope of the cochleae with implantation of PGMA or GS loaded methylene blue. Scale Bar: 1mm. **(E)** Series images of 5-wt%, 7.5-wt% and 10-wt% PGMA

loading Alizarin red S immersed in the ddH2O. **(F)** Representative SEM images of GS and PGMA. Scale bar: 50 μm.

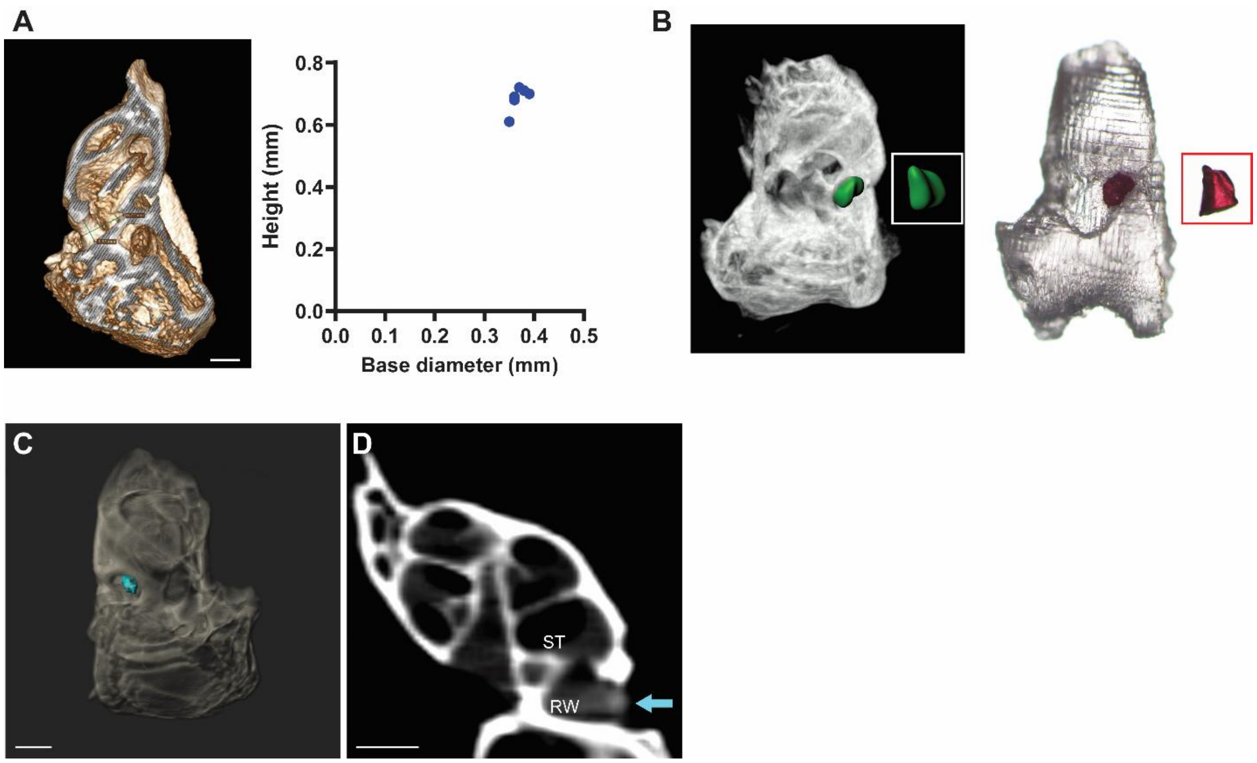

Figure S8 GS and PGMA delivery in RWM

(A) Representative micro-CT images of mice cochlea. Scale bar: 500 μm. Scatter plot represented base diameter and height of RW niche from different cochleae (n=6). **(B)** Photograph under stereo microscope of DLP 3D-printing cochlea mould. Scale Bar: 1mm. **(C and D)** Representative microCT cross-section images of mice cochlea with PGMA delivery and 3D reconstruction by Imaris with PGMA labelled in cyan. Scale bar: 500 μm. ST: scala tympani. RW: round window.

Supplemental Table

Primer sequence for qPCR analysis

Biological function	Gene name	Primer sequence	
		Forward	Reverse
OXPHOS	*Ndufb5*	CTTCGAACTTCCTGCTCCTT	GGCCCTGAAAAGAACTACG
	Sdha	GGAACACTCCAAAAACAGACCT	CCACCACTGGGTATTGAGTAGAA
	Sdhc	GCTGCGTTCTTGCTGAGACA	ATCTCCTCCTTAGCTGTGGTT
	Atp5b	GGTTCATCCTGCCAGAGACTA	AATCCCTCATCGAACTGGACG
TCA	*Mdh2*	TTGGGCAACCCCTTTCACTC	GCCTTTCACATTTGCTCTGGTC
	Idh2	GGAGAAGCCGGTAGTGGAGAT	GGTCTGGTCACGGTTTGGAA
NAD+ metabolism	*NMNAT1*	AAAGAAATCCCTAGAGCCAAAA	TCCAGCCCGAGTAACACATA
	NMNAT2	GATCCTGCTGCTGTGTGGTA	ACCACCACAATCCCAAAGTC
	NMNAT3	CACGACCCAAAAGGTTACATC	TGACAGCATCGGGAATCA
	NMNPT	ATCCTGTTCCAGGCTATTCTGT	CCCCATATTTTCTCACACGCAT
mtDNA	*Coxl*	GCCCCAGATATAGCATTCCC	GTTCATCCTGTTCCTGCTCC
	β-actin	TGTTCCCTTCCACAGGGTGT	TCCCAGTTGGTAACAATGCCA
Reference	*Rpl19*	ACCTGGATGAGAAGGATGAG	ACCTTCAGGTACAGGCTGTG
